# Preceptors’ perceptions of assessing clinical associate students at district hospital sites

**DOI:** 10.4102/phcfm.v13i1.2934

**Published:** 2021-07-12

**Authors:** Aloysious Kakia, Ian Couper

**Affiliations:** 1 Department of Family Medicine, Faculty of Health Sciences, Walter Sisulu University, Mthatha, South Africa; 2 Centre for Health Professions Education, Faculty of Medicine and Health Sciences, Stellenbosch University, Cape Town, South Africa; 3 Ukwanda Centre for Rural Health, Department of Global Health, Faculty of Medicine and Health Sciences, Stellenbosch University, Stellenbosch, South Africa

**Keywords:** preceptors, assessment, clinical associates, workplace based assessment, distributed health professionals training

## Abstract

**Background:**

Preceptors are key stakeholders in distributed health professions’ education. They supervise students in the clinical setting to enable them to have a practical experience with patients, and they assess students’ skills at the highest tier of clinical assessment. The university where this study was done conducts a distributed Bachelor of Clinical Medical Practice course on a distributed platform which is dependent on preceptors at the training sites. Understanding the perceptions of preceptors, as major stakeholders, regarding the student assessment they do will assist the faculty to provide better support and development that might be needed and assist in maximising the benefits of distributed training.

**Aim:**

The aim of this study was to explore the perceptions of preceptors regarding assessing clinical associate students at district hospitals in the Bachelor of Clinical Medical Practice programme.

**Setting:**

The study was conducted at a rural university in the Eastern Cape province of South Africa.

**Methods:**

This was a qualitative study involving nine preceptors who were purposively selected from three district hospital training sites based on their involvement in assessing clinical associate students. Semi-structured interviews were conducted, recorded, transcribed and thematically analysed.

**Results:**

Four themes emerged from thematic analysis: assessment issues, preceptor issues, student issues and university support issues. Preceptors are committed and enthusiastic in training and assessing the clinical associate students but require input from the university in terms of training and ongoing support.

**Conclusion:**

Lack of training threatens the validity of preceptor assessment. Academic institutions should train and support preceptors to enable them better to fulfil their roles.

## Introduction

Distributed training of health professionals is a strategy that could significantly contribute to improving the quality, quantity and relevance of health professionals in resource-limited settings.^1^ It involves training of students outside tertiary academic hospitals that are usually associated with universities. Such training could take place in district-level hospitals and appropriate primary healthcare facilities.^1^ Preceptors are an important stakeholder in distributed health professions education because they supervise students in the clinical setting so as to enable them to learn through practical experience with patients.^2^ Preceptorship ensures that students get individualised experiential learning opportunities, is the interface between theory and real patient management and provides for role modelling.^3^ Omer et al. include assessment as one of the four key roles of preceptors in addition to the roles of protectors, educators and facilitators.^4^ Because preceptors are typically not faculty members of the training institution, they need to be trained before they start precepting students.^5^ Clinical associates (ClinAs) are mid-level medical professionals in South Africa who were introduced into the health workforce in 2011 in an effort to address the shortage of health workers in rural areas.^6^ Training is offered at the University of Pretoria, University of the Witwatersrand and Walter Sisulu University (WSU) as a 3-year Bachelors in Clinical Medical Practice (BCMP) course. Walter Sisulu University, where this study was conducted, is a rural university in the Eastern Cape province of South Africa situated in the former Transkei Homeland. Here, ClinA training is conducted on a distributed platform that is spread over five hospitals. The students spend 75% of the training time at these hospitals during each of the three years of study, and they acquire attitudes, knowledge and skills under the preceptorship of a multidisciplinary group of health professionals including, *inter alia*, doctors, ClinAs, nurses and dieticians in the wards, outpatient department (OPD) and casualty units. The preceptors assess the students with regard to clinical skills, knowledge and professionalism as part of continuous assessment with both a formative and summative function. Clinical procedures performed by students are scored by the preceptors in a logbook on a scale of 1–5, 5 being an excellent performance of the procedure. The logbooks are specific to the year of study and contain all the procedures that the students are expected to perform during the academic year. Students also receive a score from the preceptors at the end of each ward rotation with regard to professionalism, knowledge, attitude and skills. The score for continuous assessment comprises 60% of the final mark at the end of each academic year and the preceptor assessment contributes 30% of continuous assessment. The other components of continuous assessment include written tests for every module taught, objectively structured clinical examinations, patient-based case reports and scores from class presentations and tutorials. A student must score at least 50% in continuous assessment in order to be allowed to do the end of year exams. Preceptors are therefore an important part of student assessment in the BCMP programme.

**FIGURE 1:**
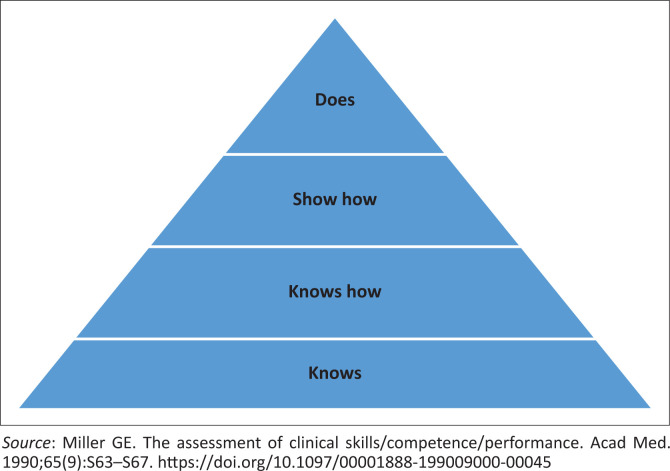
Framework for clinical assessment.

Assessment by preceptors is an example of workplace-based assessment (WPBA), which is considered to be one of the best ways of assessing competence in the health professions.^7^ Miller^8^ proposed a classification of methods of assessment in health professions education into a pyramid with four hierarchical levels as a framework within which assessment could be viewed. At the apex of Millers pyramid is assessment of performance *in vivo*. This is done by direct observation of students in real-life settings and forms the basis of assessment in the workplace. In the BCMP programme at the university under study, preceptors assess students performing tasks in the clinical setting with real patients. Their role, therefore, matches the highest tier of Miller’s pyramid for clinical competence, as depicted in Figure 1, as it is the ‘does’ that is being assessed.

The literature on preceptors’ perceptions and experiences of assessment centres around five key areas: the experience of making decisions to fail underperforming preceptees, the preceptor–preceptee relationship, support for preceptors in their role as assessors, preparation for the role of an assessor and the tools used in assessment.

The difficulty in making decisions to fail underperforming preceptees pervades the literature. A number of preceptors feel that giving a fail mark to a student means that they (the preceptors) have failed to provide an appropriate learning environment, use effective facilitation strategies and provide adequate feedback to the student, making them look incompetent as preceptors.^9,10^ The reluctance to fail underperforming students also emanates from not wanting to cause friction in the relationships,^11^ not getting the required managerial support with the decision^12^ and subjectivity on the part of the preceptor.^13^ Luhanga et al.^14^ are concerned about this reluctance to fail underperforming students because preceptors also have a role of protecting the public by preventing incompetent health professionals from becoming registered.

Conflict is a common experience in the preceptorship relationship. All the sample of Mamchur and Myrick^15^ reported having experienced conflict with preceptees. However, the majority of the preceptors in this study also reported that the conflict was later fully resolved. Meyer^13^ found that a key source of conflict was a result of preceptors acting in the dual roles of both mentor and assessor, and the conflict was accentuated when a student failed.

In the research by Palermo et al.,^16^ preceptors felt that they had not been sufficiently prepared for the assessment role. In the absence of formal training, the preceptors developed their skills from peers, student feedback, interacting with university staff members and their past experiences as students. Novice nurse preceptors in Malaysia used terms like ‘disappointment’, ‘nervous’, ‘burden’, ‘unprepared’, ‘stressful’ and ‘worry’ to describe their experiences as preceptors mainly because they were not prepared for the preceptorship role.^17^ Helminen et al.^18^ highlight the need for preceptor and faculty staff to meet in the beginning of the clinical practice period so as to have a common understanding of the assessment that will take place at the end. The literature underlines the importance of training of preceptors for them to fulfil their roles in the face of various challenges they face. They need training in conflict management, performance evaluation and as­sessment, clinical teaching strategies, formulating constructive feedback and how to match pedagogy to learning styles.^19^ Burch^20^ emphasises the undebatable need to train preceptors for effective WPBA. This not only enhances the quality of assessment, but also contributes to faculty development in primary healthcare settings and improves patient management. Botma et al.^21^ provide insight into a programme that has been used by two universities to train nurse preceptors in South Africa.

Preceptors prefer a portfolio style of assessment over tools that require ticking checklists because they perceive the former as being student-led, student-owned and having the ability to facilitate reflective practice and self-evaluation and to document student progress. The checklists were perceived by preceptors not to capture competence sufficiently, especially when assessing professional attributes and behaviours like communication, negotiation, time keeping and leadership skills in the workplace. Norcini and Burch^22^ provide a review of some instruments that are used for assessment of health professionals in the workplace, which are particularly useful in formative assessment. These include the Mini-Clinical Evaluation Exercise, Direct Observation of Procedural Skills, Clinical Work Sampling, Blinded Patient Encounters, Case-Based Discussion, Multisource Feedback and Clinical Encounter Cards.

The literature does not feature preceptor assessment of ClinAs or similar cadres. Furthermore, whilst the ClinA training programme has existed for over 10 years in South Africa, the perceptions and experiences of the preceptors who are involved in assessment have not been described. This was an important gap in knowledge because ClinAs are a new cadre in South Africa being trained in a novel learning platform (distributed sites) away from the traditional tertiary hospitals. Knowledge of their perceptions and experiences could contribute significantly to quality improvement efforts and to optimising preceptorship in the BCMP programmes in South Africa. The aim of this study therefore was to explore the perceptions of preceptors regarding assessing ClinA students at district hospital training sites in the Eastern Cape province of South Africa.

## Research methods and design

### Study design

This study was conducted at a rural university in the Eastern Cape province of South Africa in the former Transkei Homeland. It was a qualitative study that adopted an interpretivist approach and used individual interviews to collect data.

### Study population and sampling strategy

A sample of nine preceptors was purposively selected from three hospital training sites. Three participants were selected from each of the hospitals. Participants were preceptors who had been active in assessing ClinA students irrespective of the duration of time they had been preceptors. Preceptors who had been involved in teaching but not assessing ClinA students were excluded. The participants were identified by the onsite WSU tutors and the clinical managers at the hospitals. Out of the five sites used by the university for the BCMP programme, one was omitted because it is based at a regional referral hospital and another one was omitted because the primary researcher is the university tutor in charge of the site.

Potential participants were given information about the study and those who agreed to participate provided written consent. Ten preceptors were thus approached in February and March 2018 of whom nine agreed to participate. These participants are here numbered according to hospital (A, B, C) and the chronological order of interviews at the hospitals. Five doctors and four ClinAs were interviewed. Participants A1, A2, B2, C2 and C3 were doctors, whilst A3, B1, B3 and C1 were ClinAs. They included two female and seven male participants. The period the participants had been involved in training ClinAs ranged from seven months to six years, with the average for doctors being three years and for ClinAs being one year.

Semi-structured interviews were conducted on the same day by the first author using a guide which was developed from themes that emerged from the literature. All interviews were conducted in a private atmosphere to ensure confidentiality. The average duration of the interviews was 10 min. The interviews were audio recorded with written permission of the participants, and transcribed verbatim by a third party. The transcripts were reviewed by the researcher, compared with the audio recording and corrections were made where necessary to ensure accuracy.

### Data analysis

Data were analysed using the six steps of thematic analysis as described by Braun and Clarke,^23^ namely, familiarisation with the data, generating initial codes, searching for themes, reviewing themes, defining and naming themes and producing the report. The data were assigned initial codes which were open to modification as the analysis proceeded. Categories were developed from the codes that had a relationship with each other. These were further built into subthemes and themes. The initial themes were further revised into the final themes which the researcher has defined and given distinct names. The process of data analysis was regularly shared with two peers involved in medical education research and practice to enhance validity.

### Ethical considerations

Ethical approval to conduct the study was obtained from the Stellenbosch University Health Research Ethics Committee (HREC) (ethical clearance number: S17/09/180). The study was registered on the research website of the Eastern Cape Department of Health (ECDOH). Verbal permission to interview the preceptors was provided by the clinical leadership of the hospitals. Written informed consent was obtained from all the study participants prior to participation. To assure confidentiality, no names were used in the recordings and transcriptions. Each of the interviews was allocated a code which was used for the saved audio recording and the transcript. Participants in this report are referred to by these codes.

## Results

### Themes and categories

Four themes emerged from thematic analysis of the data, with several subthemes and categories. The themes were conduct of assessment, student-related issues, preceptor-related issues and university support issues, as summarised in Table 1.

**TABLE 1:** Themes and categories formed from the data.

Theme	Conduct of assessment	Student-related issues	Preceptor-related issues	University support issues
Sub-themes and categories	Venues of assessment Tools of assessment ■ Type of tools used ■ User friendliness of tools Reliability of assessment Ease of assessment	Experiences with poorly performing students Conflict with students	Preceptor skills for assessment ■ Preceptor training for assessment ■ Source of skills for assessment Satisfaction derived from assessment Preceptor motivation	University support for assessment

### Conduct of assessment

The theme of conduct of assessment included the venue of assessment, tools used in assessment, the reliability of assessment and perceptions on the ease of assessment.

Assessment is conducted in the emergency department, the OPD and the wards. It is done during ward rounds, after-hours on-call time, patient presentations and routine consultation with patients. Students are assessed for clinical knowledge, history taking, examination skills, patient assessment and management and procedural skills:

‘Well basically [I] am involved in assessing the clinical [associate] students based on the clinical skills during the hospital rounds and everything.’ (B1, ClinA, Feb 2018)

‘Mostly it’s the clinical approach to assessing patients and history taking, correct examination and how to do a procedure, and the management of the patient.’ (C2, Doctor, March 2018)

The preceptors use the assessment tools in the students’ logbooks:

‘I assess them using the procedure logbooks, checking their skills in performing clinical procedures and also check their history taking skills using the log books.’ (B3, ClinA, Feb 2018)

The preceptors also talked about the user-friendliness of the tools and the extent to which they are satisfied with them. Participants who were satisfied with the assessment tools described them as easy to use, friendly and quite helpful. Two of the ClinAs indicated that the tools were easy to use because they were trained using the same tools:

‘Very friendly to me since I was taught or trained with the same book.’ (B3, ClinA, Feb 2018)

One participant indicated that if they had any issues with the tool, they would sort it out at the beginning of the year with the help of the university staff:

‘They are usually easy to use but if we have questions we would have tackled questions right from the beginning of the year because, like I said, the [*university*] staff they come to discuss it.’ (A1, doctor, Feb 2018)

The tools are not only easy to use but also make assessment easy:

‘… The aspect of assessment that we do is purely driven by their log book so there is good direction and a good ummm, how should I say ummm …what you expect it to be, you just look at the book and you know what you are meant to oversee.’ (C2, doctor, March 2018)

Those who were not satisfied with the tools said they were too simplistic, give a narrow range of possible scores, are inaccurate and subjective. Some indicated that they would prefer the tools to be more detailed:

‘They usually have just simple questions like satisfactory, excellent, poor, not a very big range of answers that you could give.’ (C2, doctor, March 2018)

‘Checklist would be a very helpful tool, like in the OSCE there is something of that sort that would help to make it easy for you to score the student according to how they did this and how they did the other and all that. But then you see if you had to score just in percentages, like 80% but you don’t know how you got to that 80%.’ (C1, ClinA, March 2018)

Participants talked about the extent to which they found assessment to be easy or difficult. There were mixed opinions across the sites in this regard. Those who found assessment easy attributed this to the use of logbooks, enthusiastic students, students who perform well and having a small number of students:

‘I don’t think it’s a difficult task mainly because the aspect of assessment that we do is purely driven by their log book so there is good direction.’ (B2, doctor, Feb 2018)

‘It’s not that difficult to assess them because some of them they are always present and active, eager to learn those procedures, so it gets easy to assess those who attend regularly …’ (A3, ClinA, Feb 2018)

Those who found it difficult felt that there were too many students, and that students had a tendency to demand marks that they did not deserve:

‘Assessing them hasn’t been easy at all, there are so many of them.’ (C2, doctor, March 2018)

‘It’s difficult some times because you get the students who always prepare to work with you; so they are always with you; so they kind of expect favors when it comes to giving them marks … And they think that even regardless of how they performed the skill you have to give them a higher mark.’ (C2, doctor, March 2018)

Concerns over the reliability of assessment were raised by three participants. One participant stated that the assessment was prone to subjectivity:

‘I find that it’s very subjective, it really depends on the individual you are dealing with and the kind of person that you are. … For instance, if someone is my friend, although it shouldn’t happen, it does happen to tell you the truth. Telling someone to give someone marks between one and five, if you don’t like them in a certain way you lean towards the other way.’ (B2, doctor, Feb 2018)

### Student-related issues

The preceptors talked about issues in assessment that related to students. This included experiences with poorly performing students and conflict with students.

Dealing with poorly performing students evoked negative emotions amongst the preceptors which they described as ‘feeling bad’, ‘sad’, ‘annoying’ and ‘discouraging.’ It made the preceptors feel that there was something they did not do right during the teaching and provoked feelings of guilt. Preceptors said that it was hard to fail a poorly performing student:

‘I always feel bad giving low marks knowing that as someone who has been teaching these students, probably there is something that I missed out for this particular student or something wasn’t clear. It’s hard, it’s hard failing any student …’ (B1, ClinA, Feb 2018)

‘It’s a sad moment, especially if you are examining candidates that you have been teaching for a long time. So, seeing that somebody you have taught has failed, for me I feel bad. For me as a teacher, it’s my own. As far as [*I*] am concerned if I teach you, you must pass; so if somebody I have taught comes and fails, it’s sad.’ (A3, ClinA, Feb 2018)

Some preceptors make every effort to ensure that the students do not get a low mark:

‘You don’t want to give a student a low mark, … That’s why I say we help them when they are doing the procedures and all that, so that like they can understand. Because we can’t have them scoring very low marks.’ (C1, ClinA, March 2018)

‘Like you said the poorly performing students they give them another chance, okay and most of the time they improve.’ (A1, doctor, Feb 2018)

There were preceptors, however, who put the blame of poor performance squarely on the shoulders of the students and did not feel responsible. They used words like ‘careless’, ‘did not bother’ and ‘skip classes.’

‘It’s so annoying if I can put it that way but you have to, like, fail the student because they became too careless.’ (B1, ClinA, Feb 2018)

Some preceptors reported having no challenges with assessing the poorly performing student:

‘I don’t get any challenges assessing poorly performing students. Yeah, I don’t get challenges at all.’ (C3, doctor, March 2018)

Participants were asked about their experiences regarding conflict with students in the process of or as a result of assessment. The responses ranged from no experience of conflict at all to efforts to avert conflict to conflict that was attributed to various causes.

One participant said they take steps to avert conflict by giving feedback and discussing the assessment with the students, thus taking the student along the journey of learning and assessment:

‘No [*conflict*], and the reason is not that we have taken a paternalistic attitude to it. But because of the fact that when you assess them especially after a skill or after presentation, the best time to assess them is immediately after and when you give them to discuss, you give it to them, what they feel about this, and most of the time they don’t argue, there has never really been a conflict. Because we take them along.’ (A1, doctor, Feb 2018)

Another participant reported that the students identify with him as a ClinA and this reduces the chances of conflict because it becomes easier for them to discuss their concerns:

‘I think for me as a clinical associate it’s not much of a challenge because they, I think they have this perception that we are approachable. They may at a certain point get scared of the doctors but us as the clinical associates they are more open with us. So, if you take a student and sit them down wherever the challenge is and help, yes, they are easy to open up.’ (C1, ClinA, March 2018)

Where there has been conflict, it has been attributed to students’ expectations of the assessment process. Some students, for example, expect to be assessed even if they have been absent from the clinical experience:

‘Sometimes I do experience challenges especially some students they just get absent, they don’t come to OPD, they may come once or twice and you are expected to assess and yet you don’t even know that student because they don’t always go to OPD.’ (B3, ClinA, Feb 2018)

They also ask the assessors for more marks than the preceptors feel they deserve during an assessment, leading to conflict:

‘Yes. Students will ask you to please give them a higher mark and you say no. … You see tears and frustrations but, in the end, it has to be fair if someone is to pull-up their socks if they are lagging behind.’ (C1, ClinA, March 2018)

‘It’s difficult sometimes because you get the students who always prepare to work with you … so they kind of expect favors when it comes to giving them marks … And they think that even regardless of how they performed the skill you have to give them a higher mark.’ (A3, ClinA, Feb 2018)

### Preceptor-related issues

The theme of preceptor issues includes preceptors’ skills for assessment, satisfaction derived from assessment and motivation for assessment.

All the preceptors reported that they had had no formal training for assessing the ClinA students:

‘We were never trained on what to exactly check and how to assess the clinical associates. … We were never trained; the university is not involved in teaching us how to assess the students.’ (B3, ClinA, Feb 2018)

In light of this lack of training, participants employed various means of making up for the lack of training, which included relying on their own inherent abilities, previous experience and training, receiving a few hints that are built on over time and their experiences as students, as shown in the following quotes:

‘Just from my knowledge of what I was supposed to do.… It just comes naturally.’ (A1, doctor, Feb 2018)

‘Fortunately for me I was once a teacher. … So … scoring is not that much of a problem for me.’ (C1, ClinA, March 2018)

‘It’s just that someone shows you that this is how you do and you just keep following, it’s like you are in-serviced, something like that, not exactly formal training.’ (B1, ClinA, Feb 2018)

‘I have been there before. I knew what was expected of me as a student at the time when I knew that I had to do this course properly in order to get a mark.’ (A3, ClinA, Feb 2018)

With regard to the satisfaction from assessment, most participants reported that assessment is satisfying and gave various reasons: it was a reflection of the work they have been doing with the students, it is part of their duty and is an honour, assessment improves teaching skills, some students perform exceptionally well and students love the preceptors’ teaching and assessment, as shown in the comments below:

‘Yeah, I do get satisfaction from it because what really culminates into assessment is what you have done for them over some weeks or over some days.’ (A1, doctor, feb 2018)

‘I can say it’s quite a fulfilling experience because the reason as to why I was transferred here was for me to find clinical associate students to train and to assess, so I have been very honored to be involved in that.’ (B1, ClinA, Feb 2018)

‘I feel very happy being involved in training of clinical associates [*mmm*] since [*I*] am a clinical associate myself. So, hmmm, I feel like it’s also [*a*] learning curve for myself because it reminds me of what I used to do as a student and I have always met very active students who are always eager to learn and they want to know the procedures in the hospital so I feel very happy.’ (B3, ClinA, Feb 2018)

‘I was assessing, I think it was for a procedure this time, and the way he was so fine and I was like ‘this student is doing better than myself as a professional’ you see. But then I was not jealous but I was so proud, it’s so rare to find such a student, someone who is dedicated, you get to see how dedicated a child is and how hungry they are for this. …. You see that this student is really very good and if by chance you have the opportunity to assess such, it makes you feel good.’ (A1, doctor, Feb 2018)

‘Getting students who would like to work with you because they love how you teach them or, yeah, they love how you teach them and assess them.’ (A3, ClinA, Feb 2018)

### University support issues

The theme of university support issues describes the efforts by the university staff or relevant university department towards helping the preceptors to perform assessment of the ClinA students. In this regard, the preceptors’ responses show a heterogeneity across the sites. Preceptors from hospital A indicated that the support was sufficient:

‘They do support us adequately because from time to time they come to brief us, to lecture us the trainers basically. They give us tips on assessment and everything, what to look for and what not to look for periodically.’ (A1, doctor, Feb 2018)

‘They are supportive because they always tell us that we should assess the students thoroughly and with no favors because they really need to know if they are up to standard with the practical work.’ (A2, doctor, Feb 2018)

Preceptors from hospitals B and C indicated that the support was not sufficient or was absent all together.

‘They don’t interact at all, hardly interact. When they are having the tutorials we are never there, because we have our own things to do in the hospital.’** (**B2, doctor, Feb 2018)

‘Not a lot actually. They just believe that as a good doctor and as a reliable person you will do a good job … it’s a trust process more than support. Fortunately, we have not had a bad bunch of students, so it has been easy flowing.’ (C2, doctor, March 2018)

Preceptors felt that they do not know what the university expects of them and they needed the university to make this clear to them.

‘It’s not only the procedure books. There are those end of year, end of term assessment that we have to do. … They may seem straight to the point, but we need to know how we are supposed to assess the students, like university’s part of how they like us to do the assessment.’ (A2, doctor, Feb 2018)

The preceptors gave several examples of how they would want the university to support assessment. These mainly centred on formal training and the presence of university staff members during assessment:

‘So, if ever we could get probably a day or a few hours training of telling the doctors of how to properly assess …’ (A2, doctor, Feb 2018)

‘[*B*]ut I feel that if for example we are going to assess students, we can have like just a formal sit down with a person from the university, someone who understands how it works so that we can also be clear on what to look for.’ (B1, ClinA, Feb 2018)

## Discussion

A majority of the preceptors reported never receiving formal training from the university to conduct assessment of ClinA students. As a result, they depended on a variety of sources for the skills of assessment. This is similar to what was found by Palermo et al.,^16^ Enrico et al.^17^ and Blitz et al.^24^ The paucity of training has left assessment to the creativity of the preceptors, and as a result, there are differences in understanding, interpretation and appreciation of the assessment tools and assessment process as a whole, leading to lack of uniformity in student assessment across the training sites. The assessment that has a summative purpose should be conducted by trained assessors because it is at high stake. Burns^3^ states that preceptors need to be familiar with the training curriculum of the course the students are doing, in addition to the evaluation tools that are being used.

Ongoing support from the university varies by site as seen from the preceptors’ narratives. Whilst the university staff members at hospital A are in constant touch with the preceptors, the staff in hospital B are reported not to interact at all. Some informal support exists at hospital C but that too is considered insufficient. This result is indicative of a lack of uniformity in implementation across the sites, and is different from that reported by McCarthy and Murphy,^25^ who found a homogeneous lack of support. Arising from this lack of support, there was a cry for help from the preceptors. They wanted help to do a better job. They wished to understand what the university expects from them, to be trained in assessment and to have university staff with them during at least some of the assessments. This is similar to the findings of Blitz et al.^24^ who noted that emerging preceptors of medical students at distributed training sites in South Africa harboured a desire to have a closer relationship with the university, including receiving feedback on how well they were doing. Van Schalkwyk et al.^26^ consider it the responsibility of the university to support preceptors at distributed training sites, including providing them with opportunities for faculty development geared towards making them better teachers and ensuring that they receive communication that boosts confidence and makes them feel supported. Child et al.^27^ present ongoing university support for preceptors as a vital good practice.

The participants’ responses highlighted issues of reliability of the assessment. Suskie^28^ notes that the possible sources of error in student assessment include the student, the assessment instrument, the assessment environment and the rater. The results highlight each of these areas as a possible source of error. Both A1 and B2 alluded to the assessment not necessarily being a true reflection of the abilities of the students, because the students could be affected by the environment of assessment. Some preceptors indicated that the instruments do not properly capture the skills of the students. Preceptor B2 indicated that subjectivity on the part of the rater is likely to be high in the assessment of ClinA students. The raters (preceptors) are not trained to assess and lack the knowledge of what is expected of them; therefore, assessment has been left to their individual creativity, interpretation and discretion. This is compounded by reports of preceptor reluctance to fail poorly performing students and students demanding for higher marks than what they deserve. All these factors working together bring into question the reliability of preceptor assessment in the BCMP programme at the university and this echoes the need for rigorously assuring reliability in the WPBA settings.

The relationship between the preceptor and preceptee is critical to the success of preceptorship. Foley et al.^29^ state that the formation of positive working relationships between the student and preceptor determines the success of preceptorship. Cuncic et al.^30^ describe it as ‘a critical component of teaching.’ Similar to the findings of Meyer^13^ and Mamchur and Myrick,^15^ some preceptors in this study experienced a strain in the preceptor–preceptee relationship emanating from conflict. The conflict arises from the demand for higher marks than what is deserved as seen in the case of students who expected a high mark just because they spent a lot of time with preceptor A3. Mechanisms that the preceptors have used for warding off conflict included allowing students to discuss the assessment outcome, and fostering a good environment which provides a platform over which difficult issues can be discussed. Preceptors who are ClinAs found it relatively easy to build this bridge.

To the benefit of the students and the BCMP programme as a whole, the absence of training is compensated for by two factors: the enthusiasm and commitment of the preceptors, and the simplicity of the tools. This explains why the preceptors have been able to conduct assessments over the 10 years of the programme with minimal university support. Motivated preceptors are imperative for successful implementation of distributed training as asserted by De Villiers et al.^31^ The responses of the preceptors in this study show that they are motivated towards assessment and desire that it should be done better. The intrinsic nature of the motivation is indicative of a potential for longevity. The satisfaction amongst the participants also echoes the findings of De Villiers et al.^31^ who reported that preceptors at distributed training sites benefitted through greater job satisfaction and positive impact of the students on the preceptors, amongst other things. The preceptors in this study got satisfaction from seeing students grow and perform well in assessments, being loved by the students, improvement of skills and seeing the fruit of the teaching that they have been doing. The preceptor motivation and satisfaction seen in this study are important factors in the success of teaching and WPBA in the BCMP programme.

## Conclusion

This study sought to understand the experiences and perceptions of preceptors with regard to assessing ClinA students at district hospitals in the BCMP programme at the university under study. Whilst the preceptors are enthusiastic and highly motivated to teach and assess the students, the university has not actively prepared and supported them to perform these roles. The lack of preparation and ongoing support impinges on the reliability of preceptor assessment. This underscores the need for active engagement of preceptors by academic institutions. It is, therefore, recommended that institutions that engage preceptors in training and assessing students in distributed health professionals’ education should ensure initial training and ongoing support for the preceptors. A programme similar to the nurse preceptor training programmes reported by Botma et al.^21^ that are carried out at two South African universities would be useful in improving workplace-based learning and assessment. We further recommend that institutions pay attention to factors that affect the reliability of preceptor assessment in distributed medical education so as to optimise the benefits of workplace assessment environment.

### Study limitations

This study involved one programme in one academic institution. This limits the extent to which the results may be applied to other institutions. The authors have, however, made an effort to place the findings into the broader picture of the literature on assessment in clinical settings. The first author is a lecturer of the BCMP programme at WSU. This could potentially affect the interpretations that the researcher made from the data.
